# Medical Robotics in Bone Fracture Reduction Surgery: A Review

**DOI:** 10.3390/s19163593

**Published:** 2019-08-18

**Authors:** Long Bai, Jianxing Yang, Xiaohong Chen, Yuanxi Sun, Xingyu Li

**Affiliations:** State Key Laboratory of Mechanical Transmission, Chongqing University, Chongqing 400044, China

**Keywords:** medical robot, surgical robot, orthopedic surgery, fracture reduction, computer-aided surgery, computer-assisted surgery

## Abstract

Since the advantages of precise operation and effective reduction of radiation, robots have become one of the best choices for solving the defects of traditional fracture reduction surgery. This paper focuses on the application of robots in fracture reduction surgery, design of the mechanism, navigation technology, robotic control, interaction technology, and the bone–robot connection technology. Through literature review, the problems in current fracture reduction robot and its future development are discussed.

## 1. Introduction

There is an increasing trend in various types of fractures including accidental fractures caused by vehicles, high-altitude falling fractures caused by infrastructure construction, wound fractures in overweight and seniors, etc. [1]. The annual incidence of accidental (unintentional) non-fatal falls is 2831/100,000 [1], and epidemiological studies also show a worldwide incidence of 9.0–22.8/1000 per year for fractures [2].

Taking limb fractures as an example, the usual treatment process is as follows:

(1) Preoperative scan to analyze the fracture and determine the treatment plan accordingly;
(2) Aligning the fracture segment;
(3) Diagnosing the reduction situation;
(4) Fixing the aligned fracture segments with internal/external fixation;
(5) Postoperative recovery.

Traditionally, the reduction of milder fracture is achieved by manual reduction combined with external fixation [3,4], while the more serious fracture repair treatment is achieved by open surgery [5] combined with internal/external fixation. However, these traditional methods have the following disadvantages:

(a) Low accuracy, as it is largely dependent on doctor experience;
(b) The manual reduction method requires strong physical strength from the surgeons;
(c) Large external fixation frame is not convenient for postoperative recovery;
(d) Time-consuming during the workflow;
(e) Open surgery causes greater trauma and would damage soft tissues and blood supply, which would lead to a high margin of nonunion and delayed union [6].

To overcome the above drawbacks, minimally invasive techniques have been applied in fracture reduction surgeries, which have been proven to have high union rates (between 90% and 99%), and low rates of infection [7,8]. However, high X-ray exposure towards both surgeons and patents during the surgery is one of the biggest disadvantages. It could be as long as 66–414 s (about 2.9 mSv–18.2 mSv radiation dose) [9,10].

Robots are widely recognized as potential solutions to overcome the drawbacks of traditional surgeries and have quickly become the focus of current research [11,12,13,14,15,16]. As Gosling T. et al. pointed that the robot-assisted fracture surgery has the feasibility and necessity [17,18], as robot plays a significant role in assisting the surgery:

(a) High precision;
(b) Large force/torque;
(c) Low effect of radiation;
(d) Time reduction during the workflow.

An extensive survey is presented in Reference [19], which acknowledged the efficacy, safety, and superiority of applying all types of robots in orthopedic surgeries. In Reference [20], the orthopedic robot is divided into the joint surgery robot, the fracture reduction robot, the spinal surgery robot, and the trauma orthopedic robot, while in Reference [21], the orthopedic robot is summarized as different systems of computer-assisted orthopedic surgeries (CAOS) in total hip arthroplasty. However, there is little research specifically in fracture reduction treatment robots. The fracture reduction treatment is different from other orthopedic surgery such as joint replacement. Therefore, the fracture reduction robot will be explored to further enhance the perceptions of readers in its developing trend.

A great number of related studies on fracture reduction robots, especially its role in fracture reduction surgery, was reviewed for further study in this paper. Focusing on the mechanical structure, the surgery performance and the existed problems, this paper compared and summarized related research and contributions, and indicated the developing trend.

To provide sufficient literature survey, the authors searched “bone robot,” “Fracture Reduction Robot,” “orthopedic surgery robot,” “computer assisted orthopedic surgery,” in Google Scholar and got more than 48,000, 18,000, 17,000, 27,000 results, respectively. The authors then selected about 400 articles of general relevance through the following process:

(1) Ranked the results by relevance (use an algorithm provided by Google);
(2) Divided the past 15 years into three periods, and selected the top 2% articles related to each keyword within each period;
(3) Excluded those articles which do not include “robots” or “medical devices” (e.g., literature that performed case analysis, describing surgical improvements) with brief review.

After reading about 400 abstracts, 40 highly relevant documents were selected for intensive reading. To further broaden the coverage of this paper, the author also conducted interviews and consultations on experienced surgeons. At the same time, the authors reviewed the relevant citations and citing literature of the above 40 pieces of literature, and finally obtained additional 60 research papers related to fracture reduction robots.

The rest of this paper is structured as follows: the current fracture reduction robots were compared according to their different structure types in Section 2; assistive technologies were summarized in Section 3; challenges and developing trends in current status were analyzed in Section 4, and conclusion in Section 5.

## 2. The Structure of Fracture Reduction Surgery Robot

Lower limb fractures accounts for 1/3 of the total number of fractures [22]. Large forces and torques are required during its reduction surgery, especially for femoral fracture. Gosling T. et al. [23] showed that the ultimate force and torque in femoral fracture reduction surgery reached 411N and 74 Nm, respectively. Therefore, the advantages of robots are particularly prominent in lower limb fractures reduction surgery. 

The fracture reduction surgery is a procedure involving all operations required for fracture treatment, that is, the reposition of the fracture segment and the fixation after the reduction. Correspondingly, current robots under research can be divided into the reposition robot (the reduction robot) and the fixed robot (the positioning robot [24,25,26,27]). The fixed robot mainly plays an intraoperative positioning function, such as intramedullary nail fixed distal locking pin positioning, etc., which is similar to robots for joint replacement and spinal pedicle screw implantation. The reposition of broken bones, however, is the major procedure before fixing in the fracture surgery [28]. Some scholars pointed out that accurate anatomical reduction is a crucial step for the operative treatment of fractures. Failure to realign the fracture site would adequately result in the delayed union, malunion, or nonunion [29,30,31]. Therefore, the research of the reposition robot is the key technique of the fracture reduction surgery robot system. At present, there are mainly four types of structures in the reposition robot:

(a) Based on the external fixed frame structure;
(b) Based on the serial structure of the industrial robot;
(c) Parallel structure;
(d) Serial-parallel hybrid structure.

In addition to the above four types, there are also some other types of reduction robot. For example, the transform based on the traction bed [32,33,34], the sleeve-type fracture segment reducer based on a soft airbag [10,35].

### 2.1. Robot Based on the External Fixed Frame Structure

**Figure 1:**
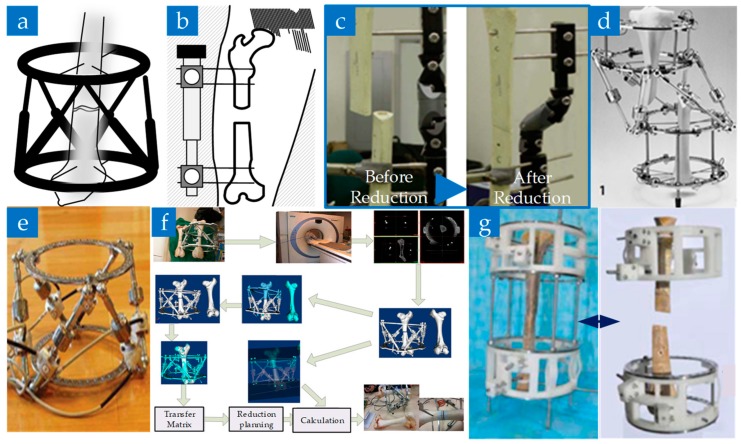
External fixator and external fixator-based fracture reduction robot. (**a**) A 6 degree-of-freedom (DOF) external fixator. (**b**) A unilateral type of external fixator. (**c**) Bone alignment before and after the application of the computer-aid external fixator [37]. (**d**) Computer-aid hexapod external fixator [38]. (**e**) A hexapod robot external fixator [40]. (**f**) A novel 3D hexapod robot and the fracture reduction procedure of using it [43]. (**g**) A 3D printed, customized external fixator [47].

The External fixed frame has two main structures, one is the Stewart six-degree-of-freedom (DOF) platform in Figure 1a, and the other one is a unilateral fixator [36] in Figure 1b.

In traditional clinical practice, the surgeon has to manipulate the fracture site to realign the proximal and distal fragments [37] by using the external fixator combined with the two-dimensional images provided by a C shape arm. While for the computer-aided system, the process will change as follows: firstly, the position of the external fixator required for resetting is obtained by the software, and then the final reduction is realized by manual adjustment. For example, the study in [37,38] (Figure 1c,d) used the DynafixTM unilateral external fixator [39] to complete the reduction with the aid of a computer.

The robot should have the ability of automatic adjustment. Seide K. et al. [40] added electromotor elements to a manually controlled fixator to realize all six spatial degrees of freedom movements, as shown in Figure 1e. The robot can obtain the initial pose and the length of each pole of the target pose by the forward and the inverse kinematics calculation, thus enabling the surgeon to control all incremental degrees of freedom movement by simply clicking the mouse.

In 2009, Majidifakhr K. et al. [41] proposed a 6-degree-of-freedom reduction robot based on the Stewart platform. Based on that, Tang P. et al. [42,43,44] applied the technology of three-dimensional reconstruction of CT scan images to improve the robot, as shown in Figure 1f. To further improve the ease of operation, Han W. et al. [45] researched the handle operation used on the same platform. In order to further improve the surgical effect, sensor-based position/force hybrid control [36] and optical tracking based navigation technology [46] are also studied and applied to external fixed-frame robots based on the above studies. With the development of the 3D printing technology, Feng Q. et al. [47] combined it with the computer-aided reduction technology to develop an external fixator with individual customization advantages, as shown in Figure 1g.

Such external fixed-frame-structure based robots have simpler structures and lower manufacturing cost. However, the operation of the reduction surgery is complicated, and a large trauma may be caused by the connection of the external fixator.

### 2.2. Robot Based on the Serial Structure of the Industrial Robot

**Figure 2:**
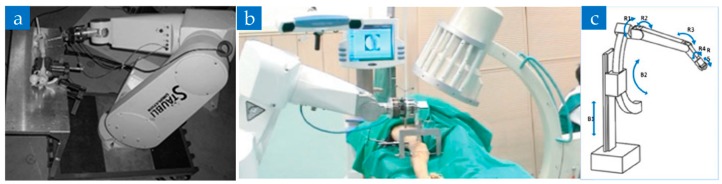
Various serial structure orthopedic trauma surgery robot based on an industrial robot. (**a**) RepoRobo designed by Regensburg Clinical University [48]. (**b**) Long bone fracture reduction robot system studied by R. Westphal et al. [8]. (**c**) An orthopedic robot with circular prismatic joint [53].

In 2004, researchers in Regensburg Clinical University of Regensburg, produced a fracture reduction robot based on the Stäubli RX 130 industrial robot, RepoRobo [48], which used a two-finger gripper and a six-dimensional force-torque sensor to ensure no slippage or plastic deformation, and have a reasonable range of force applied in surgery, as shown in Figure 2a. In 2006, Westphal R. et al. [8] transformed the Stäubli RX 90 industrial robot to a handle controlled reduction robot, which used a 2D image for navigation, as shown in Figure 2b. By 2009, they integrated a C-arm, an imaging system, a surgical navigation system, a bone reduction robot, a robot control unit, and control computers to be a long bone fracture reduction assisted robotic system [49].

Oszwald M. et al. [50,51] also adopted the Stäubli RX 90 industrial robot structure and experimented on mice and human bones to prove the feasibility. Ruan Z. et al. [52] further used the above system to perform 144 robotic-assisted fracture surgery experiments on human bone specimens without soft tissue. However, its test condition is far away from the clinical environmental requirement. In addition to this, Kuang S. et al. [53] designed a high precision structure that combines a circular prismatic joint with five passive/active back-drivable joints, which can be used for orthopedic trauma surgery such as positioning drilling, as shown in Figure 2c.

This type of serial reduction robot has the advantages of large motion space and good flexibility. However, because of its large operating space, the robot is prone to collide with other equipment and medical personnel. Besides this, its carrying capacity is relatively poor as each arm in the system is serially connected. Therefore, there is fewer studies in simple serial structure reduction robot in recent years.

### 2.3. Robot based on the Parallel Structure

Due to the need for large force and torque in fracture reduction surgery, in 2006, Graham A. E. et al. [54] first proposed a six-degree-of-freedom parallel platform for the fracture surgery, elaborating a complete surgical system concept and its advantages. Based on it, Yu L. et al. [55] used clinical experience to determine the force required for the reduction of the tibia and femur fractures, according to which the 6-PTRT (6 indicates six degrees of freedom; P indicates Prismatic-Pair; T indicates Hooke-Pair; R indicates Revolute-Pair) parallel robot with large output force (reach to 700N) and large workspace (300 mm × 160 mm × 160 mm) was designed.

**Figure 3:**
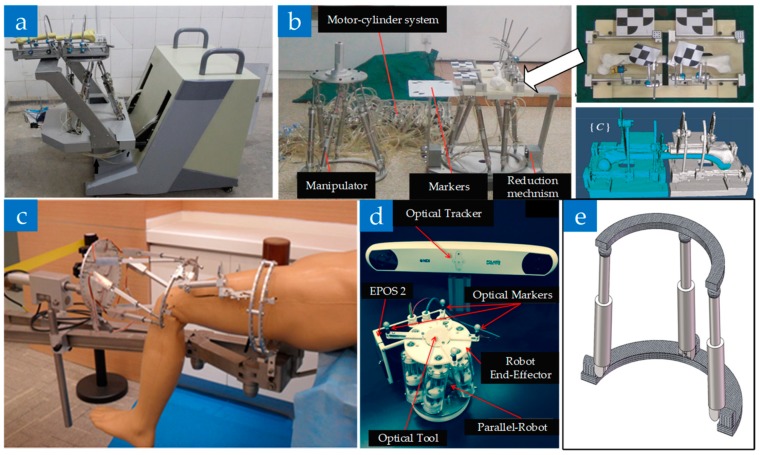
Parallel fracture reduction robot. (**a**) Stewart based closed diaphyseal fracture reduction robot [58]. (**b**) master-slave teleoperation robot [60]. (**c**) Parallel manipulator robot [61]. (**d**) Parallel fracture manipulation robot [63]. (**e**) A prototype of the wide-open robot.

Another typical structure of the parallel reduction robot is the Stewart platform. Tang P. et al. [56] designed a robot fixed at the distal of the fracture segment based on this structure, which can reduce the occupation of the operating space by using the screw driver to control the movement of the hydraulic cylinder. To prevent liquid leakage, the robot was further improved to reset the mechanism beneath the patient with a mobile platform [57,58,59], as shown in Figure 3a. Further, a novel master-slave teleoperation robot was proposed by Li C. et al., which enabled surgeons to adjust the reduction’s velocity and path during the surgical procedure [60], as shown in Figure 3b.

In addition, Wang J. et al. [61] transformed the original complete upper and lower rings into 2/3 rings to achieve convenient installation, as shown in Figure 3c. The mechanism fixed the proximal to the traction bed by bone needles, and its feasibility is experimentally verified on the cadaver bone. In 2015, Dagnino G. et al. [62,63] proposed a miniaturized parallel robot for small fractures of the neck of femur, which connects the fracture segment through only one metal bone needle with a limit load of 360 N and 12 N·m, as shown in Figure 3d. In 2017, Abedinnasab M. H. et al. [64] proposed a novel parallel wide-open robot, which consists of three legs. Each leg is actuated by a rotary and a linear actuator, as shown in Figure 3e. This robot is easy to install and has more workspace to act compared to a similarly sized Stewart platform.

Research has mainly focused on parallel robots in recent years. The parallel robots have demonstrated the characteristics of large static stiffness, high positioning accuracy, large load/weight ratio, and good stability. However, parallel robots also have a limited range of motion and need comprehensive structural designs and clinical layouts.

### 2.4. Robot Based on the Serial-Parallel Hybrid Structure

**Figure 4:**
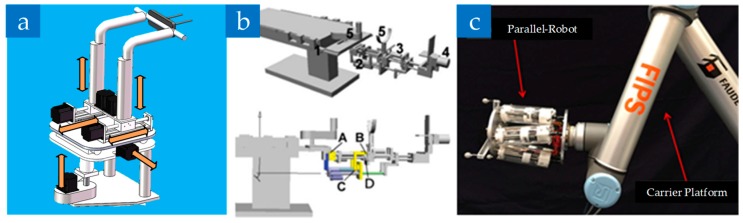
Serial-parallel hybrid fracture reduction robot. (**a**) The D’cros design and its prototype. (**b**) The serial-parallel hybrid fracture reduction robot which is mounted onto the operation table [69]. (**c**) The parallel robot connected to the robotic carrier platform (UR10) [63].

Due to the smaller range of motion of parallel mechanisms, researchers gradually adopted the serial-parallel hybrid design, which can combine the advantages of both the series and the parallel mechanisms. In 2009, Ye R. et al. [65,66,67,68] first proposed a 6-degree-of-freedom serial-parallel hybrid reduction robot (Named D’cros: Dual Cartesian robot), which obtains all the end effector rotational motion through the linear motion of the paired actuators, as shown in Figure 4a. Compared with the Stewart structure, the robot has a larger range of workspace and a simpler solution of forwarding and inverse kinematics. Further, Hung S.S. et al. [69] used a C-shaped caliper to fix the serial-parallel hybrid robot on the operating bed for the lower limb fracture assisted surgery, which can separately control of the proximal end and the distal end, as shown in Figure 4b.

In addition, Dagnino G. et al. [63,70] connected the Stewart platform to a serial robotic arm and developed a reduction robot for joint fractures, as shown in Figure 4c. Compared with most of the robots above for long bone fractures, this is a breakthrough that can reduce the trauma of patients and the damage to soft tissues, nerve vessels, etc. Even though its accuracy is high in animal bone experiments, its achievable load force is comparatively small, which restricts its practicability. Similarly, Yan et al. proposed a robot formed by a 5-DOF industrial serial robotic arm combined with a 3-DOF parallel structure end. The biggest advantage of this system is that it can perform active or passive acts based on the degree of danger in the work environment and the surgeon’s experience.

### 2.5. Comparison of Different Types of Reduction Robots

The comparison and conclusion are as follows:

(a) The reduction robot based on the external fixed frame structure was first studied because of its lowest transformation cost, but its robotic operation in the form of a unilateral fixed frame is not good.
(b) The serial robot: is almost no longer studied after 2012 due to their obvious disadvantages.
(c) The parallel robot: some were developed from Stewart-structured external fixed frame robot. With the development of navigation technology, the parallel robots are gradually combined with other equipment/technology to form a complete fracture surgery robot system. The parallel structure is also the optional structure that is used to form a more comprehensive fracture reduction surgical robot system.
(d) The serial-parallel hybrid structure: has gradually become the mainstream of research in this field due to its advantages.

The comparison of different types of reduction robots are listed in Table 1. The data of “robot size,” “maximum load”, and “application” was collected from the general survey, showing that reduction accuracy and motion range are the best results achieved by each type of robots. Although the range of motion of the parallel robot is small, the force and torque provided during the operation can meet the medical requirements. Although the experimental results of serial-parallel hybrid robots listed in Table 1 are good, its performance in long bone fractures is currently not satisfactory. Such robots in the typical literature [64,65,66,67] are still assisted by additional traction beds due to the insufficient traction they can provide. Serial-parallel hybrid robots can combine some of the advantages of series and parallel. However, most studies were based on application requirements and gave up some of the less important advantages, reducing the research difficulty.

## 3. The Assistive Technologies of Fracture Reduction Surgery Robot

**Figure 5:**
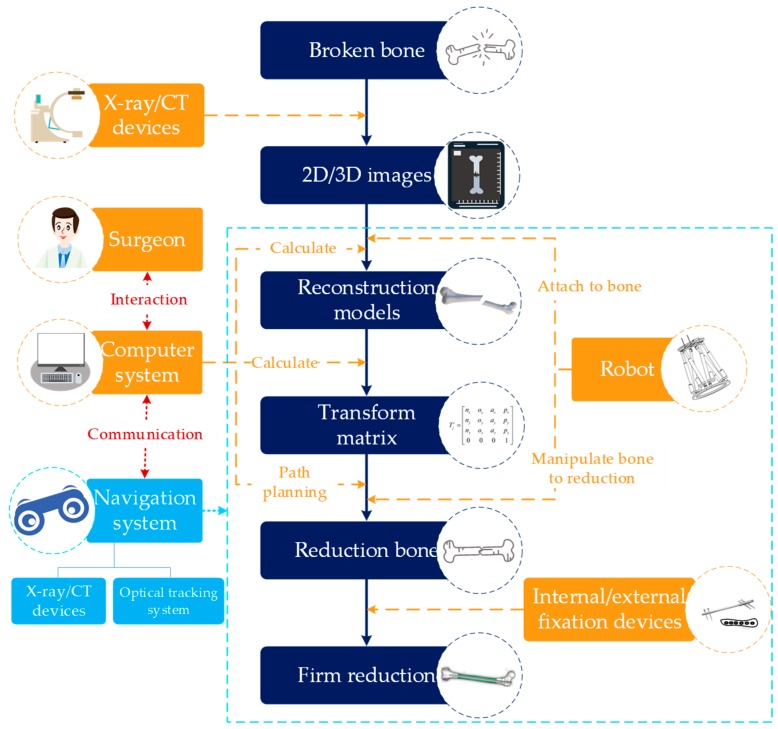
The fracture reduction robot surgical process.

In order to realize assisted fracture reduction surgery, it needs to integrate positioning robot, image acquisition equipment, a navigation system, an interactive system, etc. The typical representative is the intelligent minimally invasive surgical robotic system for the femoral shaft fracture designed by Tang P. et al. [71,72,73] and Du Z. et al. [27]. Based on the complete robot system, the robotic surgery procedure is as follows, as shown in Figure 5.

(1) A scanning diagnosis of the preoperative fracture site is performed, then a three-dimensional image of the reconstructed fracture segment is obtained by processing data, and the coordinate system information between the robot and the fracture segment is obtained by the navigation system.
(2) Calculating the transformation matrix of the robot’s current pose to the target pose through computer algorithm. Then, the path planning is carried out according to the surgical principle.
(3) The robot is automatically or controlled by the surgeon to conduct the reduction.
(4) Effective fixing is necessary after the above operations. It may be slightly different in different systems.

Based on the above process, it can be found that

(a) The navigation and the path planning technology play a decisive role;
(b) It can realize low radiation during surgery;
(c) Good interaction (for example, controlling a robot just by clicking a mouse) can help the surgeon complete the surgery more comfortably;
(d) A key issue in robotic orthopedic surgery is the bone–robot connection problem, which involves the size of the wound and the reliability of robotic procedure.

### 3.1. Navigation Technology

The role of navigation technology in trauma orthopedics is to obtain (1) the patient data via medical image acquisition devices, (2) the spatial position and posture data of the robot, and (3) the data of surgical instrument through spatial coordinate tracking device. Finally, the above data is processed by the computer system to realize the unification of the bone–robot–computer system, thereby guiding the surgeon to perform accurate and rapid fracture reduction operation.

At present, the navigation methods of orthopedic surgery mainly include: CT navigation [74,75], 2D/3D perspective navigation [76,77], ultrasound navigation [78], electromagnetic navigation [79,80], imageless navigation [46,81,82], etc. The comparison of navigation technologies is listed in Table 2.

(a) CT navigation: can obtain the high-precision tomographic data of the bone tissue. By the image processing technology, the fracture site can be reconstructed and displayed by a visual image. However, CT scanning is a relatively high-dose procedure [74].
(b) Perspective navigation: uses C-arm, G-arm, or O-arm for image acquisition, which is of high real-time performance. Compared to the 2D perspective, the 3D perspective navigation can provide preciser pre-operative planning and 3D visualization during surgery [83], which has been widely used in surgery. However, due to the volume effect, the three-dimensional image obtained is less effective than that obtained by the CT.
(c) Ultrasonic navigation (based on ultrasound imaging): is an emerging technology, the biggest advantage of which is non-invasive. However, it may be interfered by different factors, such as ultrasonic speed, distance, tissue deformation, etc.
(d) Electromagnetic navigation: besides being non-invasive, the biggest advantage compared to optical tracking is that it is not limited by occlusions of the visual field. However, it will be affected by the surrounding electromagnetic fields and metal medical equipment.
(e) Imageless navigation (optical tracking): refers to the establishment of a virtual representation of a surgical object by determining different anatomical structures and reference marks via a photoelectric tracking system. However, in minimally invasive surgery for fracture reduction, the reference marker will cause extra trauma to the patient.

Among the above navigation technologies, ultrasound navigation, electromagnetic navigation, and imageless navigation are also commonly used as external coordinate tracking devices. They are sometimes combined with the CT scanning and the perspective technology, etc. to unify the bone–robot–computer system.

To achieve more precise control, calibration of the robot is researched to study the exact position of end-effector in the camera space [71]. Through such a navigation system, the doctor can accurately manipulate the robot under guidance. However, to achieve automatic reduction, image registration is also required. At present, there are mainly two types of registration strategies: one is based on anatomical statistics; the other one is based on the image of the contralateral bone [44,84]. Within the two methods, it uses the bone model data in the statistical database or the mirror image of the reconstruction model data of the contralateral side as the reduction criterion, and use the proximal and distal images of the fracture obtained by CT scanning to solve the reduction transform matrix.

### 3.2. Robot Control and Interaction Technology

The major advantage of the fracture reduction surgery robot is its effective reduction of radiation via the remote control and automatic control. The comparison list of different control techniques is shown in Table 3 (At present, there is no real commercial fracture reduction robot product and no clinical data, so it is impossible to give an accurate data range. However, performance differences between each other can be inferred according to the principle of the control method itself).

In 1994, Bouazza–Marouf first used a robot to perform the location and drilling operation in the fracture surgery under the surgeon’s remote control [85]. The teleoperation in which mainly includes the joystick-based and the master structure-based control methods. Joystick-based control is a 2D control method, which can only move the object within an image plane. R. Westphal et al. [8,49] first used the joystick to realize the translational and rotational motion of fractured bone. The biggest benefit of the joystick-based remote operation is its low cost and short learning curve, while the disadvantage is its lack of tactile feedback [86]. Besides this, Li C. et al. [60] proposed a tactile feedbacked structure with a more direct master–slave correspondence compared with the joystick-based control, which can simplify kinematic calculations.

The automatic control is based on the relative transformation between states of the distal fracture segments. Its reduction effect depends on the preoperative planning, the reduction algorithm [84,87,88], and the accuracy of the robot itself. In reference [89], several algorithms for femur fracture reduction were compared and concluded, indicating that automatic control is more accurate than the remote control. Considering some complicated fractures, automatic control is convenient and accurate to achieve fine motion. However, automatic control relies heavily on detailed preoperative planning and does not respond well to unexpected intraoperative situations.

To make it easier for surgeons to control robots, many scholars have also studied human–computer interaction. Most human–computer interactions are using the computer interface to directly output images during the navigation and the path planning process, thereby enabling surgeons to adjust or re-plan surgery path. Su Y. et al. [90] introduced the somatosensory interaction into the fracture reduction robot, which can enable the operator to use the gesture to control the robot in the fracture reduction operation experiment.

### 3.3. Bone–Robot Connection Technology

Since the robotic fracture reduction surgery is a minimally invasive procedure, the bone–robot connection cannot be performed by a large contact tool such as a large rongeur. However, abandoning large areas of contact may reduce the strength of the connection, namely, how to fully clamp the bone to complete the reduction is still a difficult problem [72].

The commonly used bone–robot connection methods are:

(a) The external fixation pin or the screw connection;
(b) The connection through the foot boots.

Unilateral external fixator method which is used in References [48,65] is connected by a single cortical external fixation needle. The circular external fixation method in [61] was modified into an incomplete circular structure by an external fixation needle. The 6-PTRT robot [55] is connected to the patient through a needle that runs through the ankle. Moreover, Thomas Gösling et al. [49] bolted through the bone condyle and combined a single Schanz screw to connect the robot to the distal end of the fracture segment, resulting in a more robust structure. Li, C. et al. [57] fixed bone needle to a ring, and the ring is fixed to the chassis connected to the robot. Weber–Spickschen et al. tested three different robot–bone connect methods by using a conventional External Fixator, Reposition-Plate, and Three-Point-Device [91], and concluded that the last one is the only method which was able to withstand the torque needed in the reduction surgery.

Connection through external fixation pin or screw will cause 2 or more wounds, and the solid bone–robot connection generally requires a 3-point connection to avoid the bone needle and the screw from loosening, that is, at least three wounds would be caused, and iatrogenic damage may occur due to the certain cutting of the bone by screws.

Although an innovative gripping device [62] proposed by Giulio et al. using an orthopedic pin to connect bone and robot only causes one incision and one bone hole, it is only suitable for joint fractures and needs to be customized. In terms of the connection through the foot boots [32,33], the connection is achieved via the soft tissue, which can effectively reduce the wound. However, this connection is “soft connection,” which cannot achieve the solid joint of the bone–robot, and it is difficult to accurately perform the force and torque conduction. Further, it is a cross-articular fixation for the femoral fracture, which increases the reduction difficulty and reduces the stability. Thus, this method is only suitable for some operations that require less force and torque. In addition, there are other connection technologies that are constantly evolving, such as the connection of airbags [10,35], percutaneous connection techniques, etc. However, these cannot really achieve a good bone–robot connection, and there is still a long way to go in clinical experiments. Typical examples of different bone–robot connection techniques are listed in Table 4 for analysis and comparison, where the ultimate load is the maximum value that can be achieved by the bone–robot connection method.

## 4. Discussion

### 4.1. Current Difficulties in Fracture Reduction Robot Research

Great progress has been made in medical robot research in recent years, but research into fracture reduction robots has been comparatively slow. At present, there is no real fracture reduction robot for clinical use. Because the model bone, the animal bone, or the cadaver experiment is quite different from real clinical cases, a lot of robots cannot perfectly meet the clinical requirements. There are two major potential factors hindering the development of fracture reduction robots:

(a) The lack of consideration in realistic biomechanical characters. The human biomechanical model is very complicated. Although Du Z. et al. [92] established a robot model via geometry and dynamics analysis, and Wang M. et al. [93] proposed a tissue dual mechanical model, their studies are only limited in rare cases. The current research in realistic biomechanical characters cannot provide fracture reduction surgery robots with sufficient support to carry out better path planning and other operations.
(b) Not cost-effective. Most of the fracture reduction robots in current study can only perform some simple supportive operations (some even needs the help of traction equipment), while the cost is pretty high.

While from the perspective of clinical medicine, the following problems are required to be further solved and improved by the robot: (1) High radiation; (2) Grave trauma; and (3) High physical exertion of a surgeon.

### 4.2. Trends and Conjectures

**Figure 6:**
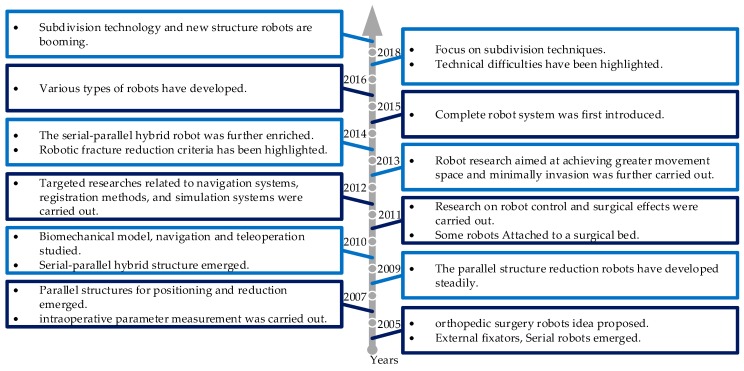
The development of research on robots for fracture reduction surgery.

The development of related research in fracture reduction surgery robot is shown in Figure 6. Until now, robots with high precision for certain needs have been proposed gradually. However, as described in Section 4.1, its advantages over traditional surgery are not completely proven via clinical trials, which restricts its practicality. From the viewpoint of the authors, fracture reduction surgery is bound to be performed via unique surgery robots. Specifically, low (no) radiation, minimal invasion (non-invasion), simple operation, and high adaptability are key factors.

(1) Low (no) radiation

Avoiding radiation damage to the surgeon can be achieved via automatic control or teleoperation. Although the current control technology has made great progress, there is still no teleoperation that can accurately reflect the intraoperative situation, where the intraoperative feedback is the most important factor for the operation. Therefore, from the viewpoint of the authors, the future control of fracture reduction surgery will be focused on achieving accurate feedbacks (include force, haptic, and so on). Besides, the authors believe that the biomechanical model study of the skeletal-muscle system will help the robot achieve accurate position/force control. 

(2) Minimal invasion (non-invasion)

The bone–robot connection will cause trauma in fracture reduction surgery. The connection between the robot and the bone that can withstand large forces and torques is usually a 3-point bone needle, while the other method that uses flexible connection is not stable enough. From the perspective of bionics, it may be a good idea, in order to achieve a stable and noninvasive connection, to mimic the human hand holding the fracture segment to move. The authors put forward two possible ways to implement a bionic connection as follows:

(a) The dexterous five-finger/three-finger manipulator that simulates the rigid-flexible combination of human hands can be used in a reduction robot to achieve a non-invasive bone–robot connection by grasping.
(b) Variable stiffness model (such as the variable stiffness device in Reference [94] achieves stiffness changes by vacuum/non-vacuum control) can be used to design the contact components of the robotic end-connecting device, and to achieve stable bone–robot connection.

(3) Simple manipulation

A simple operation is to make the robot perform the relevant surgical operation according to the doctor’s intention in a simple way. The development of the sensor technology and the interactive technology may enhance the robot’s understanding of the surgeon’s intentions. The authors indicate two main directions the manipulation of robotic fracture reduction surgery may develop to:

(a) Individuation. The operating system may consider more about the surgeons’ habits, and the hardware that is in direct contact with the surgeon may be designed as a modular part that can be personalized as needed.
(b) Virtual reality. By virtual reality technology, surgeons can operate remotely or guide surgery, and can obtain accurate surgical perception in virtual reality. In particular, breakthroughs in the reconstruction of patients’ fracture sites in virtual space will contribute to the ultimate realization of virtual reality operations.

(4) High adaptability

Most of the reduction procedures can achieve the reduction requirement in a large motion space by a serial-parallel hybrid mechanism, which may become the main configuration of future robots. However, in the femoral reduction surgery, the requirement on force and torque is higher. Currently, only the parallel mechanism’s load capacity can meet the requirements. However, it is restricted by its movement space so that it is only suitable for specific operations. With the development of research in modularization and metamorphic mechanisms, the reduction robot will be expected to have modular and variable mechanism capabilities and can be combined with various types of structures to adapt to the needs of the fracture surgery.

In terms of robotics, as far as the authors concerned, in addition to key technologies discussed above, big data technology have big potentiality in medical management and surgery [95,96], so it will be applied in this area in the future. Post-operative tracking of fracture reduction surgery will also be guaranteed based on the big data technology. Preoperative diagnosis and surgical operation data of fracture patients will be recorded, and data on recovery of postoperative bone healing will be recorded as well. A large number of patients’ surgical data will be analyzed and processed to provide strong reference support for surgeons’ future diagnosis.

**Figure 7:**
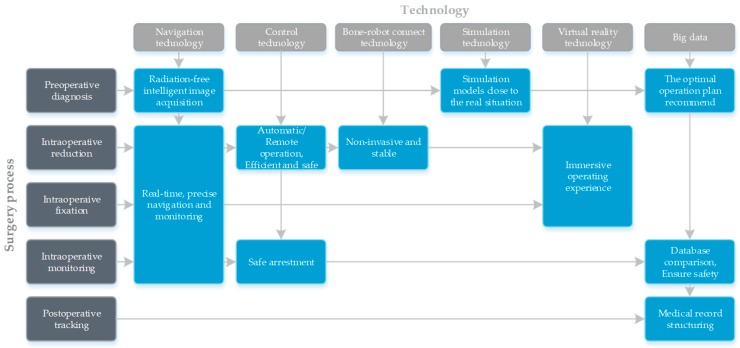
The correspondence among future robotics and fracture reduction surgery and the possible effects of future robotics on fracture reduction surgery.

The corresponding relationship among the future technologies and the fracture reduction surgery process and the possible effects are shown in Figure 7. The introduction of big data will enable robot technology to cover the whole process of fracture reduction surgery.

## 5. Conclusions

This article has reviewed the development of fracture reduction surgery robots over the past decade. The fracture reduction surgery robot is a product combining medical and engineering work, and it has made a great progress in the past few years. Various types of robots are continuously developed. According to their different structures, they can be classified into the external fixed frame structure, the serial structure based on an industrial robot, the parallel structure, and the serial-parallel hybrid structure. Among them, the serial structure has better maneuverability, and the parallel mechanism can better meet the requirements of force and torque in the reduction surgery, while the serial-parallel hybrid structure has the advantages of both the serial and the parallel structure to varying degrees.

In recent years, research into robot structure has made great progress, which meets the needs of surgery to some extent. Based on the characteristics of the robotic application in fracture reduction surgery, the research in this field has gradually focused on key technologies and problems such as surgical navigation, robot control and interaction, and bone-to-machine connection. In the navigation technology, the use of CT scanning equipment to acquire images and optical tracking devices for navigation has become the mainstream. In the robot control and the interaction technology, both the teleoperation and the automatic control have been applied. In addition, the feedback of surgical information including mechanical parameters is the main factor that affects the practicality of the surgery robot, where the instant intraoperative processing is significant in the automatic control. Regarding the bone–robot connection problem, reducing the trauma caused by stable connections is the focus. In addition, the research on skeletal model establishment and simulation system has also begun to develop in recent years, but there is still certain limitation in the research results obtained.

At present, robots used in the fracture reduction surgery have achieved remarkable results in surgical precision, robot control and surgical navigation. However, the foundation of the fracture reduction surgery robot technology is still not mature enough to provide effective support for the clinical practice. In the future, the fracture reduction surgery robot will develop toward low (no) radiation, minimal invasion (non-invasion), simple manipulation, and high adaptability. In addition, the virtual reality and big data technology will also be applied. Post-operative tracking of the fracture reduction surgery may be guaranteed based on the big data technology.

## Figures and Tables

**Table 1:** Robot comparison.

	Type	The External Fixed Frame Structure	Serial Structure	Parallel Structure	Serial-Parallel Hybrid Structure	
Comparison Item						
robot size		<leg length <2~3 times of leg diameter	general industrial robot size	>fixed frame, <serial structure	larger than the parallel structure (sometimes bigger than the serial structure)	
maximum load	force	/	<300 N	300~700 N	200~400 N	
	torque	/	/	20~80 N·m	/	
application		1/3 of the middle long bone fracture	femoral shaft fracture	femur long bone fracture	joint fracture	
Typical robot		six-bar parallel reduction mechanism [43,44]	robot in Brunswick university of technology [50]	precision surgery robot for long bone fracture [71]	serial-parallel hybrid robot (by Giulio) [70]	
test subject *		animal bone	model bone	cadaver	model bone	animal bone
reduction accuracy *	axial deflection	1.24 ± 0.65 mm	1.08 ± 0.63 mm	3.08 ± 1.505 m	1.67 ± 0.778 mm	the displacement deviation is: 0.09 ± 0.08 mm; the angular deviation is: 0.15 ± 0.04°
	rotation	2.83 ± 0.9°	1.09 ± 0.73°	2.58 ± 1.240°	2.08 ± 0.669°	
	translation	1.19 ± 0.37 mm	1.61 ± 1.23 mm	1.92 ± 0.606 m	1.33 ± 0.563 mm	
	angulation	2.34 ± 1.79°	1.37 ± 1.39°	1.98 ± 0.619°	1.50 ± 0.558°	
motion range *		120 mm × 120 mm × 80 mm	/	200 mm × 200 mm × 200 mm	serial platform: 4/3 × π1300^3^ mm^3^ parallel platform: 20.5 mm × 20.5 mm × 30 mm	

The sign “/” in the table indicates that the relevant data is not found, and the expression in the reduction accuracy column is filled based on the evaluation indexes of each robot operation, so the expressions of different robots are slightly different. The sign “*” means data from typical robots (selected the best results achieved by this type of robot), other data were obtained through general surveys.

**Table 2:** The comparison of different navigation technologies.

	Navigation	CT	Perspective	Ultrasound	Electromagnetic	Imageless
Comparison Item						
radiation		very high	high	none	none	none
visualization effect		best	better	common	common	need to combine CT images
trauma		none	none	none	none	great
disturb by environment		none	none	great	great	less

**Table 3:** The comparison of different control technologies.

	Control Technology	Joystick Based Control	Master Structure-Based Control	Automatic Control
Comparison Item				
learning curve		short	long	very short
accuracy		common	common	high
feedback ability		lack of tactile feedback	comprehensive feedback can be achieved	none
can it deal with emergencies well?		can	can	can’t

The comparison of the learning curve is based on the control method.

**Table 4:** The comparison of different bone–robot connection technologies.

	Connection	External Fixator	Reposition-Plate	Three-Point-Device	One-Orthopaedic-Pin	Through Gasbag
Comparison Item						
reliability		stable	stable	stable	stable under small external force	unstable
trauma		multiple holes	3 holes	3 holes	1 hole	none
ultimate load		<411 N; <70N·m	<411 N; <70N·m	600 N; 80 N·m	147 N; 1.8 N·m	lateral force 800 N
preoperative preparation time		>5 min	3~5 min	3~5 min	1~3 min	<1 min
customized		no	no	no	yes	no

The load that does not indicate the lateral force is the axial force and the axial torsional moment. The preoperative preparation time refers to the time needed to insert bin or assembles connecting devices.
